# Synthesis Characterization and Biological Activities of Coordination Compounds of 4-Hydroxy-3-nitro-2*H*-chromen-2-one and Its Aminoethanoic Acid and Pyrrolidine-2-carboxylic Acid Mixed Ligand Complexes

**DOI:** 10.1155/2017/6426747

**Published:** 2017-02-07

**Authors:** Temitayo Aiyelabola, Ezekiel Akinkunmi, Efere Obuotor, Idowu Olawuni, David Isabirye, Johan Jordaan

**Affiliations:** ^1^Department of Chemistry, Obafemi Awolowo University, Ile-Ife, Osun State, Nigeria; ^2^Department of Pharmaceutics, Obafemi Awolowo University, Ile-Ife, Osun State, Nigeria; ^3^Department of Biochemistry and Molecular Biology, Obafemi Awolowo University, Ile-Ife, Osun State, Nigeria; ^4^Department of Chemistry, North-West University, Mafikeng Campus, Mmabatho, South Africa; ^5^Focus Area Chemical Resource Beneficiation, North-West University, Potchefstroom Campus, Potchefstroom, South Africa

## Abstract

Coordination compounds of 4-hydroxy-3-nitro-2*H*-chromen-2-one and their mixed ligand complexes with aminoethanoic acid and pyrrolidine-2-carboxylic acid were synthesized by the reaction of Cu(II) and Zn(II) salts in molar ratio 1 : 2 for the coumarin complexes and 1 : 1 : 1 for the mixed ligand complexes, in basic media. The compounds formed were characterized using infrared, Uv-vis spectrophotometric analyses, mass spectrometry, magnetic susceptibility measurements, and EDX analyses. It was concluded that 4-hydroxy-3-nitro-2*H*-chromen-2-one coordinated as a monobasic ligand for all the complexes; it also coordinated via the carbonyl moiety in the case of the Cu(II) mixed ligand complexes. Similarly it was proposed that the amino acids also coordinated in a bidentate fashion via their amino nitrogen and carboxylate oxygen atoms. The synthesized compounds were screened for their antimicrobial and cytotoxic activities. The complexes exhibited marginal antimicrobial activity but good cytotoxic activity.

## 1. Introduction

**Figure 1:**
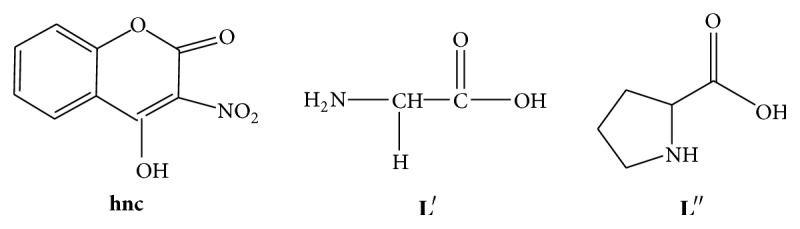
Structures of the ligands.

Coumarins are a group of oxygen heterocyclic compounds which have attracted much attention as a result of their varied pharmacological properties which include antibacterial, anticoagulants, antibiotic, antifungal, anticancer, and anti-inflammatory [1–10]. In addition, these compounds are used as additives in food and cosmetics, as dispersed fluorescent brightening agents, and as dyes for tuning lasers [11–16]. This broad array of usage has led to the increased interest in its various derivatives [9, 10, 16–18]. Of interest is the 4-hydroxy-3-nitro-2*H*-chromen-2-one derivative,** hnc **(Figure 1) [9, 10, 16, 17]. This is partly due to the presence of the carbonyl, nitro and hydroxo moieties in this compound, for which it may be considered as a versatile polyfunctional ligand [9, 10, 16, 17]. Thus it may behave as an* O*-monodentate ligand as well as an* O, O*-bidentate chelating agent [9, 10, 16, 17]. Its coordination chemistry is therefore of interest to the coordination chemist. Published reports, in which** hnc **coordinated as a neutral ligand and in others as a monobasic bidentate ligand, suggest that its coordination behaviour is a function of the pH of the reaction [9, 10, 16, 17]. In more recent times an area of focus of coordination chemists is that of mixed ligand complexes. This is because they are the most general and probable form of coordination compounds in the biological system [9, 19–22]. Therefore studies of mixed ligand complexes of biologically important compounds may serve as models for biochemical processes [20, 23, 24]. They are also characterized by their extreme stability and the fact that the chemical individuality and peculiarities of the central metal ion is more pronounced in these complexes [19].

It has been shown from previous reports that coordination of metal ions to biologically active agents may improve their efficiency and accelerate their bioactivity [25–31]. Furthermore, earlier studies have also shown that some coordination compounds of coumarin derivatives and that of amino acids exhibited promising antimicrobial and cytotoxic activities on chelation [9, 32–44]. Few studies have however been conducted on coordination compounds of pyrrolidine-2-carboxylic acid, a nonpolar amino acid, as well as mixed ligand complexes of** hnc **and amino acids. As a result of resistance to current drugs and emerging new diseases there is constant need of obtaining antimicrobial and anticancer agents with minimal side effects. The reported widespread applications of the coumarin moiety, amino acids, and their coordination compounds, therefore, informed our interest in the syntheses of novel mixed ligand complexes containing** hnc **and amino acids with the aim of obtaining more potent antimicrobial and cytotoxic agents with possible minimal side effects. Hence in this work we report the syntheses, characterization of Cu(II) and Zn(II) coordination compounds of 4-hydroxy-3-nitro-2*H*-chromen-2-one and their mixed ligand complexes with aminoethanoic acid (**L**′) and pyrrolidine-2-carboxylic acid (**L**′′), Figure 1, their antimicrobial activity, and brine shrimp lethality bioassay.

## 2. Materials and Methods

The chemicals and solvents used were of analytical grade and were used without further purification. The infrared spectra were recorded in the region 4000–499 cm^−1^ on a Fourier-Transform infrared spectrophotometer at North-West University, Mafikeng Campus. Electronic spectra were measured on a Varian Cary 50 ultraviolet-visible spectrophotometer also at the North-West University; measurements were made from 200 to 800 nm. Magnetic susceptibility measurements were carried out at room temperature in the Department of Chemistry, Kwara State University, Ilorin, using a Sherwood scientific balance with [HgCo(SCN)_4_] as standard. EDX analyses were obtained using Shimadzu Ray ny EDX 720 at the Department of Chemistry North-West University, Mafikeng Campus. The mass spectrum was obtained at the laboratory for analytical services, North-West University, Potchefstroom, on a Bruker Ser# micrOTOF-Q II 10390 mass spectrometer, using matrix assisted laser desorption ionization. Screening of the compounds for antimicrobial activity was done at the Pharmaceutics laboratory Obafemi Awolowo University Ile-Ife. Brine shrimp lethality assay was carried out at the Department of Biochemistry and Molecular Biology Obafemi Awolowo University Ile-Ife.

The compounds were synthesized using adaptation of previous reports by Creaven et al., 2005 [9]. Compound** 1** has been previously synthesized, characterized, and screened for antimicrobial activity by Creaven et al., 2005 [9]. The equations of the reactions are given in (1)–(5).

Coumarin Ternary complexes:
  (1) $$\begin{array}{c} M_{\left(aq\right)}+{2hnc}_{\left(aq\right)}⟶{\left[\;M{\left(\;hnc\;\right)}_{2}{\left(\;H_{2}O\;\right)}_{2}\;\right]}_{\left(aq\right)}, \end{array}$$

where M = Cu(II), Zn(II).

Mixed ligand complexes:
  (2) 2CuCl2·2H2Oaq+2hncaq+2L′aq⟶CuhncL′2H2Oaq
  (3) ZnCl2aq+hncaq+L′aq⟶ZnhncL′H2O2aq
  (4) 2CuCl2·2H2Oaq+2hncaq+2L′′aq⟶CuhncL′′2H2Oaq
  (5) ZnCl2aq+hncaq+L′′aq⟶ZnhncL′′H2O2aq,

where** hnc** = 4-hydroxy-3-nitro-2*H*-chromen-2-one; **L**′  = aminoethanoic acid; **L**′′  = pyrrolidine-2-carboxylic acid.

### 2.1. Syntheses of Coordination Compounds

#### 2.1.1. Compound** 1**

An aqueous solution of CuCl_2_·2H_2_O (1.72 g, 0.01 M) was added to a solution of NaOH (0.80 g, 0.02 M) and 4-hydroxy-3-nitro-2*H*-chromen-2-one (4.17 g, 0.02 M) in water and the mixture was heated and stirred on a water bath for 2 h and then cooled. A green precipitate was obtained, which was recrystallized using ethanol-water mixture (70/30 v/v), washed, filtered, and dried in a vacuum oven at 60°C to give compound** 1**, Yield: 3.99 g (78%), M.pt/d.t: 234–236°C (d). The complex was sparingly soluble in ethanol and methanol but soluble in water.

#### 2.1.2. Compound** 2**

An aqueous solution of zinc(II) chloride (1.36 g, 0.01 M) was added to a solution of NaOH (0.80 g, 0.02 M) and 4-hydroxy-3-nitro-2*H*-chromen-2-one (**hnc**) (4.21 g, 0.02 M) in water and the mixture was heated and stirred for 2 h on a water bath and then cooled. A yellow precipitate was obtained, which was recrystallized using ethanol-water mixture (70/30 v/v), washed, filtered, and dried in a vacuum oven at 60°C to give compound** 2**, Yield: 3.43 g (67%), M.pt/d.t: 245-246°C (d). The complex was sparingly soluble in ethanol, methanol, and water.

#### 2.1.3. Compound** 3**

An aqueous solution of CuCl_2_·2H_2_O (1.72 g, 0.01 M) was added to a solution of NaOH (0.40 g, 0.01 M) and 4-hydroxy-3-nitro-2*H*-chromen-2-one (**hnc)** (2.02 g, 0.01 M) in water and the mixture was heated and stirred on a water bath. A solution of aminoethanoic acid (0.75 g, 0.01 M) in ethanol-water mixture was added drop-wise; the resultant mixture was heated for 2 h. A bluish green precipitate was obtained, which was recrystallized using ethanol-water mixture (70/30 v/v), washed, filtered, and dried in a vacuum oven at 60°C to give** 3**, Yield: 3.26 g (78%), M.pt/d.t: 179–181°C (d), M^+•^(*m*/*z*): 705. The complex was sparingly soluble in ethanol, methanol, and water.

#### 2.1.4. Compound** 4**

An aqueous solution of zinc(II) chloride (2.05 g, 0.015 M) was added to a solution of NaOH (0.61 g, 0.015 M) and 4-hydroxy-3-nitro-2*H*-chromen-2-one (**hnc**) (3.31 g, 0.015 M) in water and the mixture was heated and stirred on a water bath. Aminoethanoic acid (1.13 g, 0.015 M) was added drop-wise with stirring; the mixture was then heated for 2 h. A yellow precipitate was obtained, which was recrystallized, washed, filtered, and dried in a vacuum oven at 60°C to give** 4**, Yield: 3.60 g (63%), M.pt/d.t: 249–252 (d). The complex was sparingly soluble in ethanol and methanol but soluble in water.

#### 2.1.5. Compound** 5**

A solution of copper(II) chloride dihydrate (1.71 g, 0.01 M) in water (10 ml) was added to a solution of NaOH (0.40 g, 0.01 M) and** hnc **(2.20 g, 0.01 M) in water (10 ml); pyrrolidine-2-carboxylic acid (1.16 g, 0.01 M) was added with stirring. The mixture was heated for 2 h and then cooled. Upon standing a bluish green precipitate was formed, which was filtered, washed with methanol and cold water, and then dried under vacuum at 60°C. The solid was recrystallized from water, Yield: 3.14 g (46%), M.pt/d.t.: 245–247 (d). The compound was sparingly soluble in ethanol and methanol but soluble in water.

#### 2.1.6. Compound** 6**

A solution of zinc(II) chloride (1.38 g, 0.01 M) in water (10 ml) was added to a solution of NaOH (0.04 g, 0.01 M) and** hnc **(2.09 g, 0.01 M) in water (10 ml), pyrrolidine-2-carboxylic acid (1.25 g, 0.01 M) was added drop-wise, and the mixture was heated with stirring for 2 h. Upon standing a yellow precipitate formed, which was filtered, washed with methanol and cold water, and then dried under vacuum at 60°C. The solid was recrystallized from water, Yield: 2.41 g (57%), M.pt/d.t: 280–283°C (d). The complex was sparingly soluble in ethanol and methanol but soluble in water.

### 2.2. Antimicrobial Methodology

The organisms used were five Gram-positive and three Gram-negative bacteria and two fungi, namely,* S. aureus*,* S. epidermidis*,* B. subtilis 12*,* B. subtilis 82*,* Clostridium*,* K. pneumonia*,* P. aeruginosa*,* E. Coli*,* C. albicans*, and* C. Pseudotropicalis.* The agents were dissolved in water at room temperature or hot water as appropriate to give a concentration of 40 mg/ml. The resulting solutions were used to soak sterile Whatman No. 2 discs (diameter = 6 mm) and allowed to dry in an oven at 50°C. The discs were then used to determine antibacterial and antifungal activities as previously described by Aiyelabola et al. 2012 [45]. Discs of imipenem and chlorhexidine were used as positive controls for bacteria and fungi, respectively. Zones of inhibition were used as indices of antimicrobial actions.

### 2.3. Cytotoxicity Bioassay

The procedure used was modified from the assay described by Solis et al., 1993 [46]. Brine shrimps* (Artemia salina)* were hatched using brine shrimp eggs in a conical shaped vessel (1 L), filled with sterile artificial seawater under constant aeration for 48 h. After hatching, active nauplii free from egg shells were collected from brighter portion of the hatching chamber and used for the assay. Ten nauplii were drawn through a Pasteur pipette and placed in each vial containing 4.5  mg/l of brine solution. In each experiment, different volume of sample was added to 4.5 ml of brine solution to give different concentration (20, 40, 60, 80, and 100 *μ*g/ml) and maintained at room temperature for 24 h under the light. The surviving larvae were counted. Experiments were conducted along with control (vehicle treated), of the test substances in a set of three tubes per dose. Estimation of the LC_50_ values was estimated using probit analysis on a USEPA computer program.

## 3. Results and Discussion

### 3.1. Infrared

The infrared spectra analyses of ligands 4-hydroxy-3-nitro-2*H*-chromen-2-one, aminoethanoic acid, and pyrrolidine-2-carboxylic acid and their corresponding coordination compounds were carried out and the relevant peaks (cm^−1^) are given in Table 1. The spectral assignment was achieved by comparing the infrared spectra of the ligands with that of the complexes.

#### 3.1.1. Compounds** 1** and** 2**

The spectrum of the free ligand 4-hydroxy-3-nitro-2*H*-chromen-2-one,** hnc**, exhibited a sharp band at 1522 cm^−1^, attributable to –NO_2_ asymmetric absorption stretching frequency [47]. This was shifted in the spectrum of** 1 **and** 2** to higher frequencies, thus serving as evidence of coordination of one of the oxygen atoms of the nitro group and is in agreement with previous reports on similar compounds [9]. In the spectrum of** hnc **the –OH stretching frequency was observed as an intense broad band at 3435 cm^−1^. This was however absent in the spectra of the complexes, suggesting the deprotonation of the hydroxy group. This is in accord with that observed in previous reports [9, 48]. The spectrum of** hnc** also exhibited a broad band at 2892 cm^−1^, attributable to intramolecular hydrogen bonded –OH [47, 49, 50]. This band disappeared in the spectra of the coordination compounds, indicating the breakdown of hydrogen-bonding and subsequent deprotonation of the –OH group prior to coordination of the resultant oxygen anion. This was further corroborated by the observed shifts in the *ν*(C–O) (alcoholic) stretch of** hnc **at 1009 cm^−1^ to higher energy by 48 and 62 cm^−1^ in the coordination compounds of** 1** and** 2**, respectively [47, 49, 50]. A weak broad band however was observed within the range 3468 cm^−1^ in the spectra of** 1 **and** 2 **and is suggestive of the involvement of the oxygen atom of water in coordination [49, 50]. The appearance of a new medium intense band at 1135 cm^−1^ which is a characteristic bending vibration of coordinated water molecule, *δ*(M–O–H), further confirmed this [50].

There were significant shifts in the (C=O) stretching frequency which lowered upon complexation for complexes** 1** and** 2** (Table 1) compared to that of the ligand. Previous reports have taken similar shifts as evidence of coordination via the carbonyl oxygen. However, more recent studies have suggested that this is not so. It was pointed out that such shifts may be as a result of the carbonyl being located on the same lactone ring as the ligand binding sites in the complexes [9, 10]. New bands absent in that of the ligand were observed at 637 and 621 cm^−1^ (Table 1), which were assigned as stretching frequencies of metal-oxygen bonds [48, 50, 51].

#### 3.1.2. Compounds** 3** and** 4**

The infrared spectra of the mixed ligand species of** 3** and** 4** provided evidence of coordination of** hnc **to the corresponding metal(II) ions. Such evidence was demonstrated by the shifts in the –NO_2_ asymmetric stretching frequency, observed at 1522 cm^−1^ in the free ligand but which shifted to higher frequencies in** 3** and** 4 **(Table 1), thus indicative of coordination of an oxygen atom of the nitro group [8, 10]. The deprotonation and subsequent involvement of the oxygen atom in coordination was indicated by the disappearance of the band at 3435 cm^−1^ in** hnc** in the spectra of the complexes. A new weak and broad band at 3446 cm^−1^ assignable to *ν*(O–H) frequency suggested coordination via the oxygen of water molecule. This was confirmed by the observance of *δ*(M–O–H) frequencies at 1125 and 1202 cm^−1^ for compounds** 3** and** 4 **[48, 50].

The infrared spectrum of uncoordinated** L**′ exhibited *ν*(N–H) stretching frequency at 3196 cm^−1^. The spectra of compounds** 3** and** 4 **both exhibited bands at 3301, 3263, 3374, and 3254 cm^−1^, respectively, assigned to *ν*_asy_(N–H) and *ν*_sy_(N–H) stretching frequencies and indicative of the involvement of nitrogen atom of its amino substituent in coordination of the metal ion with the ligand [50]. It should be noted that complexes** 1** and** 2** did not exhibit this stretching frequencies in comparison. The appearance of new bands, which were not present in the spectrum of the ligands, at 501 and 509 cm^−1^ ascribable to metal-nitrogen absorption frequencies of** 3 **and** 4**, respectively, further confirmed this [48, 52]. The observed medium band at 1112 cm^−1^ in the free ligand was attributed to the *ν*(C–N) absorption frequency and this blue shifted on coordination in the complexes [50]. The spectrum of** L**′ also exhibited bands at 1590 and 1480 cm^−1^ ascribed as *ν*_asy_(COO) and *ν*_sy_(COO) stretching frequencies, respectively [50]. The shifts in *ν*_asy_(COO) absorption to 1669 and 1672 cm^−1^ for** 3** and** 4**, respectively, and that for *ν*_sy_(COO) stretch to 1449 and 1466 cm^−1^ are indicative of the coordination of the oxygen atom of the carboxylate group in the coordination compounds [16, 44, 50, 53]. The hypsochromic shifts in the *ν*_asy_(COO) frequency and bathochromic shifts for the *ν*_sy_(COO) frequency for compounds** 3** and** 4** indicated the monodenticity of the carboxylate ion on coordination. This is in agreement with established reports and was confirmed by the observed energy difference (Δ*ν* = 200 − 225) between both asymmetric and symmetric stretches in the coordination compounds [16, 17, 50, 54]. New bands that were absent in the ligand but present in that of the complexes at 618 and 655 cm^−1^ (Table 1) were attributed to the M–O stretching frequencies [44, 48, 50].

#### 3.1.3. Compounds** 5** and** 6**

Similar to that obtained for complexes** 1**–**4, **the infrared spectrum for complex** 5 **suggested coordination via an oxygen atom of –NO_2_ of** hnc**; this is due to the observed shift in the –NO_2_ asymmetric stretching frequency in comparison to that displayed by the free ligand** hnc**. However in the spectrum of compound** 6** this absorption band was not observed; it is suggested to be as a result of overlap of the *ν*_asy_(COO), *ν*_sy_(COO), *ν*(C=C), and *ν*(C=O) absorption bands which all lie within the region close to that expected for the –NO_2_ asymmetric stretch [50]. Both spectra of compounds** 5** and** 6 **however exhibited broad bands within the region 3444 cm^−1^; this in comparison with the observed band at 3435 cm^−1^ in the spectrum of** hnc** also suggests the deprotonation of the –OH with probable coordination of water molecule. The (C–O) stretching frequency was observed at 1068 and 1045 cm^−1^.

In the case of pyrrolidine-2-carboxylic acid *ν*(N–H) was observed as a single band at 3152 cm^−1^ in its spectrum. This was shifted hypsochromically to 3233 and 3510 cm^−1^ for** 5** and** 6** on complexation [47, 55], thus indicating coordination via the nitrogen of the amino substituent on coordination. Supporting this further is the observed shifts to higher wave number for the *ν*(C–N) stretching frequency (Table 1). This was corroborated by the appearance of M–N band observed at 562 and 557 cm^−1^ for compounds** 5** and** 6**, respectively, which was absent in both ligands. The observed strong band at 1640 cm^−1^ in the free ligand** L**′′ was attributed to the *ν*_asy_(COO) absorption and this blue shifted on coordination in both complexes [50]. The *ν*_sy_(COO) band at 1492 cm^−1^ in the free ligand** L**′′ gave bathochromic shifts in that of the complexes. These shifts are similar to that obtained for compounds** 3** and** 4** and suggest the monodentate nature of the carboxylate moiety in coordination. The observed energy difference between both asymmetric and symmetric stretches in the coordination compounds (Table 1) corroborates this further [16, 17, 50, 54]. In addition, new bands were observed at 680 and 604 cm^−1^ and were assigned as metal-oxygen stretching frequencies for compounds** 5 **and** 6**, respectively [50].

### 3.2. Electronic Spectra and Magnetic Moment

The electronic spectrum for the free ligand,** L**′, exhibited bands at 199, 230, and 331 nm attributed as *n* → *σ*^*∗*^, *n* → *π*^*∗*^, and *π*^*∗*^ → *π*^*∗*^ transitions [47, 49]. Similarly the spectrum of** L**′′ also exhibited bands at 197 and 234 nm, ascribed to *n* → *σ*^*∗*^ and *n* → *π*^*∗*^ transitions. For the free ligand 4-hydroxy-3-nitro-2*H*-chromen-2-one intense absorption bands at 205, 220, 297, and 410 nm were observed which were assigned as *n* → *σ*^*∗*^, *n* → *π*^*∗*^, and *π*^*∗*^ → *π*^*∗*^ transitions. These transitions are ascribable to the major chromophore systems of the ligands. Shifts in these bands and the observed d-d transitions are presented in Table 2.

The electronic spectrum of compound** 1** exhibited a well resolved band at 548 nm and a weak band at 632 nm assignable to ^2^B_1g_ →  ^2^A_1g_ and ^2^B_1g_ →  ^2^E_g_ transitions, which suggested an octahedral geometry [32, 56]. This proposed geometry was corroborated by its magnetic moment of 2.60 BM, indicative of a tetragonally distorted octahedral geometry [28, 40]. This is in agreement with that proposed by previous workers for similar compounds [9, 57, 58]. Intraligand transitions were observed at 215, 281, and 392 nm. The spectrum of** 2 **exhibited a band at 705 nm ascribed to charge transfer band. No d-d absorption band was observed in the visible region for compound** 2**. A magnetic moment of zero was obtained, indicating that there was no unpaired electron for the metal ion. This is in accord with that obtained by previous workers on Zn(II) complexes [55, 56, 59].

The visible spectrum of compound** 3** displayed broad band at 668 and 720 nm assigned to ^2^B_1g_ →  ^2^A_1g_ and ^2^B_1g_ →  ^2^E_1g_ transitions. Its magnetic moment of 1.53 BM is indicative of an antiferromagnetic spin-spin interaction through molecular association with possible Cu–Cu interaction or dimerization [55]. However a dimerized geometry is proposed based on the molecular ion obtained from its mass spectrum. Similar results have been published for coordination compounds of amino acids and coumarins [18, 41]. The intraligand transition was observed at 370 nm. The spectrum of** 4 **exhibited no band in the visible region of the spectrum. This is partly due to the fully filled 3d of Zn(II) ion. Its magnetic moment of zero corroborated this further. Intraligand transition band was observed at 480 nm for this compound. An octahedral geometry is however proposed for this complex and is consistent with results obtained for the metal analysis using EDX and EDTA complexometric titration (cal.: 17.06; found: 16.84).

The visible spectrum of compound** 5** exhibited a broad band at 670 nm assignable to ^2^B_1g_ →  ^2^A_1g_ transition and a shoulder at 723 nm assigned as ^2^B_1g_ →  ^2^E_1g_ transition. Its magnetic moment of 1.42 BM is suggestive of dimerization [55]. Intraligand transition was observed as an intense band at 370 nm. The spectrum of** 6 **exhibited no band in the visible region of the spectrum. Its magnetic moment of zero corroborated this further. Its intraligand transition band was observed at 484 nm. An octahedral geometry is proposed for this complex and is supported by results obtained for the metal analysis using EDX for qualitative and EDTA complexometric titration for quantitative analysis (cal.: 15.44; found: 14.57).

**Figure 2:**
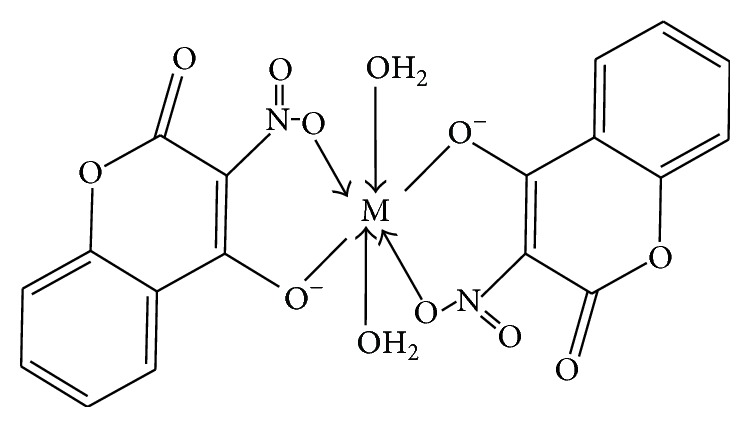
Proposed structure for compounds** 1** and** 2**.

Based on the results obtained it is suggested that compounds** 1** and** 2** exhibited octahedral geometry comprising two** hnc **ligands coordinating in a bidentate fashion and two monodentate water ligands. It is proposed that the two** hnc **ligands are coordinated to the metal ion via the deprotonated hydroxo moiety and an oxygen atom of the nitro group [9, 17, 18, 60, 61]. This result is in agreement with that obtained by Creaven et al., 2005, for similar compounds [9]. Hence the structure shown in Figure 2 is proposed for these complexes.

**Figure 3:**
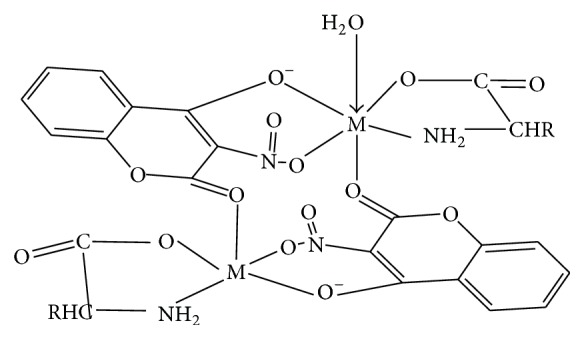
Proposed structure for compounds** 3** and** 5**.** R=–**H, compound** 3,** and –C_4_H_8_, compound** 5**.

In the case of compounds** 3** and** 5 **the results obtained indicated that coordination occurred via an oxygen atom of the nitro group and the oxygen of deprotonated hydroxo substituent in** hnc **[9, 17, 18, 60, 61]. However the carbonyl of** hnc **is proposed to coordinate with neighbouring metal ion leading to dimerization. For the amino acid, however, coordination is suggested to occur via the amino nitrogen and an oxygen atom of the carboxylate ion. According to Nakamoto, 2009, it has been shown that the oxygen atoms of the carbonyl groups which are not coordinated to the central metal ion are hydrogen bonded either to the amino group of the neighbouring molecule or to water of crystallization or are bound weakly to the metal of the neighbouring complex. It is proposed that in** 3** and** 5 **such carbonyl groups are weakly bound to the metal of the neighbouring complex [50, 62]. As a result the structure as shown in Figure 3 is suggested for the complexes. This was corroborated by the observed parent molecular ion M^+•^, obtained from the mass spectrum of compound** 3,** and is consistent with the expected molecular formula for the proposed structure.

**Figure 4:**
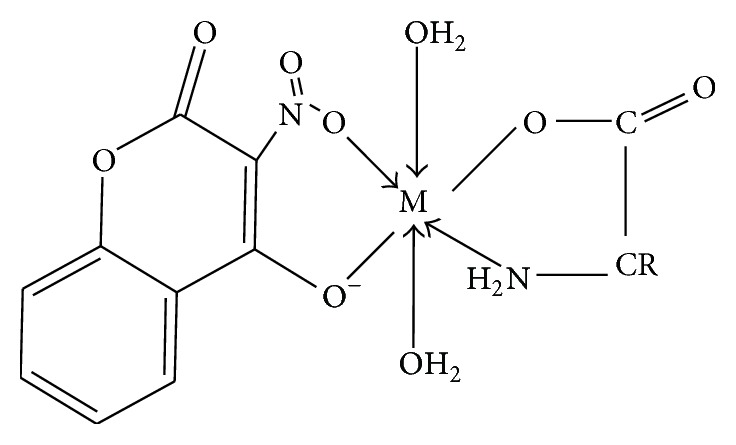
Proposed structure for compounds** 4** and** 6**.** R=–**H, compound** 4,** and –C_4_H_8_, compound** 6**.

For compounds** 4** and** 6 **the results obtained suggested an octahedral geometry; it is proposed that coordination occurred via one of the nitro* O*– atoms and the deprotonated oxygen in** hnc**. However, the amino acids coordination occurred via the amino* N*– and one of the carboxylato* O*– atoms. As a result a representation of the proposed structure of the complexes is given in Figure 4.

These results further affirmed the coordination behaviour of** hnc **as a function of the pH of the reaction. This is suggested by the deprotonation of hydroxo group in alkaline medium contrary to that obtained by previous reports for less basic media [9, 10, 18]. The results obtained also validated previous report on the enhanced individuality of the central metal ions in the mixed ligand complexes [19].

### 3.3. Antimicrobial

A comparative evaluation of the antimicrobial activity of** hnc**,** L**′,** L**′′, and the synthesized compounds** 1**–**6** was carried out against five Gram-positive bacteria, three Gram-negative bacteria, and two fungi. The results obtained are presented in Table 3. The result indicated that** L**′ and** L**′′ were both inactive to all the tested organisms. On the other hand** hnc **exhibited weak activity against* K. pneumoniae*. Both standards (imipenem for bacteria and chlorhexidine for fungi) exhibited significantly better activity than all the synthesized compounds and ligands (*P* < 0.05). Only three of the compounds demonstrated antimicrobial action and this activity was shown against only* S. aureus* and* K. pneumoniae*, Table 3. Compounds** 2**,** 3**, and** 4** exhibited good activity against* S. aureus,* a Gram-positive bacteria, suggesting the enhanced lipophilicity of the complexes on coordination [27, 28, 30, 63]. Compounds** 2** and** 3** exhibited activity against* K. pneumonia*. It would have been expected that the** L**′′ adducts should be more active against Gram-positive bacteria, as a result of the nonpolar side chain of** L**′′ with plausible enhanced lipophilicity of the complexes relative to that of** L**′, but this was not so [27, 28, 30, 63]. The reason for this is not quite evident. This therefore serves as an indication that activity does not depend solely on the ease of movement of an antimicrobial agent through the cell wall of the microbes, but rather a synergistic effects of many factors [64–66].

### 3.4. Cytotoxicity

Brine shrimp lethality assay of the synthesized compounds and ligands was carried out, and the result obtained indicated that** hnc **(LC_50_ 7.56 *μ*g/ml) was the most active of the ligands. This was followed by** L**′ (LC_50_ 8.01 *μ*g/ml) and** L**′′ (LC_50_ 11.96 *μ*g/ml). The order of activity of the compounds is as follows: compound** 2 > 1 > 5 > 3 > 4 > 6**, with LC_50_ 11.56, 12.00, 12.68, 12.93, 16.49, and 35.91 *μ*g/ml, respectively. The standard K_2_Cr_2_O_7_ (LC_50_ 5.56 *μ*g/ml) however exhibited significantly higher (*P* < 0.05) cytotoxic activity compared with that of the ligands and synthesized compounds. The synthesized compounds on the other hand exhibited significantly better activity relative to their metal salts, namely, zinc(II) chloride (LC_50_ 88.89 *μ*g/ml) and copper(II) chloride dihydrate (LC_50_ 98.34 *μ*g/ml). Similar results have been reported for cerium coumarin complexes [25]. The ternary** hnc **complexes had comparable activity in some cases relative to their mixed ligand counterparts. Thus indicating that the formation of adducts did not enhance the cytotoxic activity of the ternary** hnc **complexes. This may be attributed to the fact that the amino acids had lesser activity than** hnc**. The results obtained indicated that the zinc(II) complexes were more active than the copper(II) complexes of similar species with the exception of the pyrrolidine-2-carboxylic acid adduct. This may be ascribed to the size of the metal ion, the better activity of the metal salt, and more importantly the structure of the compounds. The activity of the pyrrolidine-2-carboxylic acid adduct of zinc, compound** 6, **suggests the nontoxic nature of the complex [67, 68]. The results suggest therefore that although chelation may not enhance the brine shrimp lethality ability of the synthesized compounds, it should be noted that it however enhanced the nontoxic nature of the ligands. Moreover, the cytotoxicity of the metal salts increased on chelation.

## 4. Conclusion

It was concluded from the study that the available coordinating species in 4-hydroxy-3-nitro-2*H*-chromen-2-one is a function of the pH of the reaction. In addition the enhanced individuality of the central metal ion in mixed ligand complexes was also demonstrated in the study. It was further concluded that although chelation may enhance the bioavailability of coordination compounds as therapeutic agents; however other factors also play important roles for their effectiveness.

## Figures and Tables

**Table 1:** Relevant infrared spectra bands for the ligands and complexes (cm^−1^).

Bands	**hnc**	L′	L′′	**1**	**2**	**3**	**4**	**5**	**6**
(cm^−1^)									
*ν*(O–H)	3435			3468	3526	3430	3446	3446	3580
*ν*(N–O)	1522			1557	1568	1560	1574	1573	
*ν*(C–O–H)	1303								
*δ* (O–H)				1207	1214	1125	1202	1211	1216
*ν*(C–O)	1009			1057	1071	1046	1151	1068	1045
*ν*(C=C)	1601			1603	1607	1605	1608	1612	1603
*ν*(C=O)	1701			1652	1668				
*ν*_as_(N–H)		3196	3152			3301	3374	3233	3510
*ν*_sy_(N–H)						3263	3254		
*ν*_as_(COO)		1590	1640			1669	1672	1667	1667
*ν*_sy_(COO)		1480	1492			1449	1466	1467	1442
*ν*(C–N)				1341	1552	1275	1380	1330	1308
*ν*(M–N)						563	565	562	557
*ν*(M–O)				637	621	618	655	680	604

**Table 2:** Electronic spectra bands (nm) for the ligands and complexes.

Compounds	**1**	**2**	**3**	**4**	d-d	*μ*_eff_
Bands (nm)						
**Hnc**	205	220	297	410		
L′	199	230	331			
L′′	197	234				
**1**				392	548, 632	2.6
**2**				460	—	
**3**				370	668, 720	1.53
**4**				480	—	0
**5**				370	670	1.42
**6**				484	—	0

**Table 3:** Result of zone of antimicrobial inhibition (mm) for the ligands and complexes.

	**hnc**	L′	L′′	**1**	**2**	**3**	**4**	**5**	**6**	**C**
*S. aureus *	—	—	—	—	09	—	17	—	—	44
*S. epidermidis*	—	—	—	—	—	—	—	—	—	34
*B. subtilis 12*	—	—	—	—	—	—	—	—	—	34
*B. subtilis 82 *	—	—	—	—	—	—	—	—	—	29
*Clostridium*	—	—	—	—	—	—	—	—	—	34
*K. pneumoniae*	09	—	—	—	—	07	08	—	—	34
*P. aeruginosa*	—	—	—	—	—	—	—	—	—	39
*E. coli*	—	—	—	—	—	—	—	—	—	33
*C. albicans *	—	—	—	—	—	—	—	—	—	36
*C. pseudotropicalis *	—	—	—	—	—	—	09	—	—	36

C means imipenem and chlorhexidine for bacteria and fungi, respectively.
